# Robotic retroperitoneal surgery in a uro‐oncology specialised centre: Translating RPLND expertise from testicular cancer to non‐genitourinary retroperitoneal tumour resection

**DOI:** 10.1002/bco2.70180

**Published:** 2026-03-25

**Authors:** Alberto Costa Silva, Jeremy Oates, Aziz Gulamhusein

**Affiliations:** ^1^ The Christie NHS Foundation Trust Manchester UK; ^2^ São João University Hospital, Faculty of Medicine of University of Porto, RISE – Health Research Network Porto Portugal

**Keywords:** lymph node excision, retroperitoneal neoplasms, robotic surgical procedures, sarcoma, testicular neoplasms

## Abstract

**Objective:**

The aim of this study is to evaluate the feasibility and perioperative outcomes of robotic‐assisted resection of selected non‐genitourinary retroperitoneal tumours (RA‐RTR) performed by urologists with established experience in robotic retroperitoneal lymph node dissection (RA‐RPLND) within a specialised centre.

**Methods:**

A retrospective analysis was conducted of all patients undergoing RA‐RPLND for testicular cancer and RA‐RTR for non‐genitourinary tumours between January 2022 and May 2025. Procedures for renal, adrenal and upper tract urothelial malignancies were excluded. All operations were performed using a four‐port da Vinci Xi® platform.

**Results:**

Forty‐five patients were included: 27 RA‐RPLND and 18 RA‐RTR. RA‐RTR patients were older (median 50 vs. 36 years, *p* = 0.024). Preoperative tumour size was larger in RA‐RTR (56 mm vs. 20 mm, *p* < 0.001), although specimen dimensions were similar. Operative time was longer for RA‐RPLND (143 vs. 90 min, *p* = 0.045). Estimated blood loss (40 vs. 20 mL, *p* = 0.077) and length of stay (1 vs. 2 days, *p* = 0.272) were comparable. No conversions or transfusions occurred. Complication rates did not differ (14.8% vs. 5.6%, *p* = 0.333), and all cases achieved negative margins.

**Conclusion:**

Comparable perioperative outcomes between RA‐RTR and RA‐RPLND indicate that these surgeries can be performed in specialised centres by urologists experienced in complex retroperitoneal surgery.

## 1 INTRODUCTION

Urologists are highly familiar with the retroperitoneum through both open and robotic‐assisted (RA) approaches, raising the question of whether this expertise can be effectively translated to non‐genitourinary retroperitoneal tumour surgery.

Retroperitoneal lymph node dissection (RPLND) for testicular cancer is one of the most complex urological procedures and shares key anatomical landmarks, dissection planes and en bloc resection principles with non‐genitourinary retroperitoneal tumour resection (RTR), providing a conceptual and technical common ground.^1^, ^2^ Traditionally, such procedures have been performed via open approaches, but there is a growing body of evidence supporting RA techniques.^3^, ^4^, ^5^

Our institution is a tertiary referral centre for retroperitoneal tumours, with combined expertise in oncological urology and retroperitoneal sarcoma surgery led by urologists, which has enabled the development of a translational surgical skillset and the adoption of RA techniques across both indications.

This study aims to evaluate the feasibility of urologists performing non‐genitourinary retroperitoneal tumour surgery by directly comparing perioperative outcomes of RA‐RPLND and RA‐RTR.

This study aims to evaluate the feasibility and perioperative outcomes of RA‐RTR performed by urologists with established experience in RA‐RPLND within a specialised centre.

## 2 METHODS

A retrospective analysis of all patients who underwent RA‐RPLND in the context of testicular cancer or RA‐RTR for non‐genitourinary tumours between January 2022 and May 2025 at a single referral centre was performed. The study was conducted in accordance with the Declaration of Helsinki and received approval from the institutional research ethics committee. Due to the retrospective nature of the analysis, the requirement for individual informed consent was waived. All patient data were retrieved from institutional clinical data and were anonymised prior to analysis to ensure confidentiality. Retroperitoneal surgeries for renal, adrenal or upper tract urothelial tumours (primary treatment or recurrences) were excluded.

RA‐RPLND was performed for post‐chemotherapy residual masses in non‐seminomatous (NSGCT) or seminomatous germ cell tumours (SGCT), as well as in selected primary cases. Indications for RA‐RTR included biopsy‐confirmed non‐genitourinary retroperitoneal malignancies, symptomatic or large lesions without prior biopsy with typical imaging features and indeterminate masses with inconclusive biopsy findings. All RA‐RPLND procedures were performed by a single urological surgeon with extensive experience in both open and robotic upper urinary tract and sarcoma surgery. The RA‐RTR procedures were undertaken either by the same surgeon or by a second surgeon, also possessing expertise in robotic surgery and open sarcoma resections. All procedures were performed using the da Vinci Xi® robotic system (Intuitive Surgical, Sunnyvale, CA, USA) in a four‐port configuration with an auxiliary AirSeal® system (CONMED, Utica, NY, USA). Patient positioning was determined by the laterality of the disease: Unilateral procedures were carried out in the flank position, whereas bilateral procedures were performed in the supine position.

Data collected included age, body mass index (BMI), Eastern Cooperative Oncology Group (ECOG) performance status, radiological measurements based on Computed Tomography (CT) or Magnetic Resonance Imaging (MRI). Intraoperative variables included operative time, estimated blood loss (EBL) and the need for adjunct procedures, while postoperative outcomes comprised length of hospital stay (LOS) and complications classified according to the Clavien–Dindo system. Histopathology results and the need for subsequent treatment were also recorded. All data were collected and analysed using the Statistical Package for the Social Sciences (SPSS), version 30.0 (IBM Corp., Armonk, NY, USA). The Shapiro–Wilk test was applied to assess the normality of data distribution. Normally distributed continuous variables are presented as the mean ± standard deviation and non‐normally distributed data as the median (percentile 25th–percentile 75th). Categorical variables are expressed as absolute values and percentages. Comparisons between groups were performed using the independent *t*‐test or Mann–Whitney *U* test for continuous variables, depending on distribution, and the χ^2^ test or Fisher's exact test for categorical variables. A two‐tailed *p* value < 0.05 was considered statistically significant. No statistical comparison was performed for variables related to disease biology (indication for surgery, prior systemic or radiation therapy, histology and postoperative oncological treatment), as these represent distinct pathologies.

## 3 RESULTS

A total of 45 patients were included, comprising 27 RA‐RPLND and 18 RA‐RTR. Baseline demographic and perioperative characteristics are summarised in Table 1. The median age was lower in the RA‐RPLND group compared to the RA‐RTR group (36.0 [27.0–43.0] vs. 50.0 [33.0–61.0] years, *p* = 0.024). There were no significant differences in BMI or ECOG performance status between groups. Prior radiotherapy occurred only in the RA‐RTR cohort (5.6%), whereas prior chemotherapy occurred exclusively in the RA‐RPLND group (70.4%).

**TABLE 1:** Comparison of baseline and perioperative characteristics between robotic‐assisted retroperitoneal lymph node dissection (RA‐RPLND) for testicular cancer and robotic‐assisted resection of non‐genitourinary retroperitoneal tumours (RA‐RTR).

	RA‐RPLND *n* = 27	RA‐RTR *n* = 18	*p*‐value
Age, years, median (P25–P75)	36.0 (27.0–43.0)	50.0 (33.0–61.0)	0.024
BMI, kg/m^2^, median (P25–P75)	30.0 (27.0–34.0)	26.0 (23.0–32.0)	0.951
ECOG, % (*n*)	0–88.9 (24) 1–11.1 (3)	0–61.1 (11) 1–33.3 (6) 2–5.6 (1)	0.073
CT biggest dimension, mm, median (P25–P75)	20.0 (17.0–28.0)	56.0 (39.0–100.0)	<0.001
Gender, % (*n*)	Male 100.0 (27)	Male 50.0 (9) Female 50.0 (9)	^a^
Symptoms, % (*n*)	‐	Pain 27.8 (5) Haematuria 5.6 (1)	0.001
Side, % (*n*)	Left 59.3 (16) Right 40.7 (11)	Left 61.1 (11) Right 38.9 (7)	0.901
Previous radiotherapy, % (*n*)	‐	5.6 (1)	^a^
Previous chemotherapy, % (*n*)	70.4 (19)	‐	^a^
Previous abdominal surgery type, % (*n*)	Appendectomy 8.0 (2) Hernia 8.0 (2)	Cholecystectomy 11.1 (2) Appendectomy 5.5 (1)	0.867
Indication, % (*n*)	NSCGT post chemotherapy: 59.3 (16) SCGT post chemotherapy: 11.1 (3) NSGCT primary PPT: 11.1 (3) SGCT primary (stage IIa): 18.5 (5)	Biopsy proven lesion: 50.0 (9) Size/symptoms: 27.8 (5) Inconclusive biopsies: 22.2 (4)	^a^

Abbreviations: BMI, body mass index; CT, computed tomography; ECOG, Eastern Cooperative Oncology Group performance status; mL, millilitres; mm, millimetres; MRI, magnetic resonance imaging; RA‐RPLND, robotic‐assisted retroperitoneal lymph node dissection; RA‐RTR, robotic‐assisted resection of retroperitoneal tumours (non‐genitourinary).

^a^ Not performed.

Intraoperative and postoperative data are presented in Table 2. In terms of indication for surgery, most RA‐RPLND cases were NSGCT post‐chemotherapy (59.3%, *n* = 16), and RA‐RTR were most frequently performed for biopsy‐proven tumours (50.0%, *n* = 9). Despite smaller preoperative lesions in the RA‐RPLND group (20.0 mm [17.0–28.0] vs. 56.0 mm [39.0–100.0], *p* < 0.001), specimen dimensions were similar (80.0 mm [52.0–93.0] vs. 65.0 mm [58.0–100.0], *p* = 1.000).

**TABLE 2:** Operative details, postoperative outcomes and histopathological findings in robotic‐assisted retroperitoneal lymph node dissection (RA‐RPLND) and robotic‐assisted resection of non‐genitourinary retroperitoneal tumours (RA‐RTR).

	RA‐RPLND *n* = 27	RA‐RTR *n* = 18	*p*‐value
Surgical time, minutes, median (P25–P75)	143.0 (119.0–168.0)	90.0 (70.0–120.0)	0.045
Template, % (*n*)	Unilateral, 92.6 (24) Bilateral, 7.4 (2)	Unilateral, 100.0 (18)	0.236
Adjunct procedures, % (*n*)	‐	22.2 (4)	0.010
		Ureteral catheterisation, 11.2 (2)
		Nephrectomy, 5.6 (1)
		Adrenalectomy, 5.6 (1)
Estimated blood loss, mL, median (P25–P75)	40.0 (20.0–56.0)	20.0 (10.0–50.0)	0.077
Drain, % (*n*)	26.6 (8)	11.1 (2)	0.143
Length of stay, days, median (P25–P75)	1.0 (1.0–2.0)	2.0 (1.0–3.0)	0.272
Complications, % (*n*)	14.8 (4)	5.6 (1)	0.333
Clavien–Dindo, % (*n*)	I: 3.7 (1); ileus	I: 5.6 (1); foot drop (transient)
	II: 3.7 (1); pancreatitis	
	IIIa: 7.4 (2); chyle leak with drain insertion	
Specimen largest dimension, mm, median (P25–P75)	80.0 (52.0–93.0)	65.0 (58.0–100.0)	1.000
Final histology, % (*n*)	Teratoma, 55.6 (15) Viable seminoma, 22.2 (6) Viable mixed NSGCT, 11.1 (3) No tumour, 7.4 (2) Teratoma with somatic transformation, 3.7 (1)	Sarcomas^a^, 16.8 (3) Haemangioma, 11.1 (2) Schwannoma, 16.8 (3) Paraganglioma, 5.6 (1) Lymphangioma, 11.1 (2) Lymphoma, 16.6 (3) Solitary fibrous tumour, 11.1 (2) Ovarian fibroma 11.1 (2)	^b^
Need of further treatment after surgery, % (*n*)	3.7 (1); seminoma patient with viable tumour in RPLND specimen with retroperitoneal recurrence after surgery and submitted to BEP3	16.7 (3); chemotherapy for lymphomas	^b^

Abbreviations: BEP3, bleomycin, etoposide and cisplatin (three cycles); mL, millilitres; mm, millimetres; NSGCT, non‐seminomatous germ cell tumour; RA‐RPLND, robotic‐assisted retroperitoneal lymph node dissection; RA‐RTR, robotic‐assisted resection of retroperitoneal tumours (non‐genitourinary); SGCT, seminomatous germ cell tumour.

^a^ Including leiomyosarcoma, liposarcoma and spindle cell sarcoma.

^b^ Not performed.

Estimated blood loss was 40.0 (20.0–56.0) mL in the RA‐RPLND group compared to 20.0 (10.0–50.0) mL in the RA‐RTR group (*p* = 0.077). There was no significant difference in the LOS between groups, and there were no cases requiring blood transfusion or conversion to open surgery. Adjunct procedures were less frequent in the RA‐RPLND group compared to the RA‐RTR cohort (0% vs. 22.2%, *n* = 4; *p* = 0.010). Surgical time was longer in the RA‐RPLND group (143.0 [119.0–168.0] min) compared with the RA‐RTR group (90.0 [70.0–120.0] min, *p* = 0.045). Drain placement was more common in the RA‐RPLND group (26.6%, *n* = 8) than in the RA‐RTR cohort (11.1%, *n* = 2), although this difference did not reach statistical significance (*p* = 0.143).

The overall complication rate was not significantly different between groups (14.8% in RA‐RPLND vs. 5.6% in RA‐RTR, *p* = 0.333). The most common histology in the RA‐RPLND group was teratoma (55.6%, *n* = 15), whereas the RA‐RTR cohort demonstrated a heterogeneous range of tumour types.

All achieved negative surgical margins. Further treatment following surgery was required in 3.7% of RA‐RPLND (one seminoma case with viable tumour managed with chemotherapy) and 16.7% of RA‐RTR patients (all with lymphoma requiring chemotherapy).

## 4 DISCUSSION

For surgeons trained in retroperitoneal urologic oncology, particularly RA‐RPLND, many of the technical steps in RTR—retroperitoneal access, vascular control and en‐bloc dissection—are familiar.

Our centre has extensive experience in complex retroperitoneal surgery, both open and robotic, including nephrectomies with or without IVC thrombectomy, adrenalectomy, and nephroureterectomy. These procedures have refined our expertise, which translates to the management of non‐genitourinary retroperitoneal tumours. We used RPLND as a reference procedure in this study because, despite histological and biological diversity, the surgical exposure, anatomical planes and technical demands overlap substantially between RA‐RPLND and RA‐RTR.

In our series, peri‐operative metrics (length of stay and complications) were comparable in both cohorts. The observed age difference between groups aligns with the known age distribution of the respective tumour types. Similarly, the higher rates of preoperative systemic therapy in the RA‐RPLND cohort reflect its common indication in this setting. Such prior treatment can also render the procedure more technically challenging due to the fibrosis associated with post‐chemotherapy residual masses. Adjunct procedures (nephrectomy, adrenalectomy or ureteral catheterisation) were required in 22.2% of the RA‐RTR, likely reflecting the local invasiveness of non‐genitourinary retroperitoneal tumours. Margin status is a key concern in RTR, where incomplete excision has prognostic implications.^6^, ^7^ In our cohort, all cases achieved negative margins, reflecting patient selection—specifically limited tumour volume and the absence of requirement for multivisceral resection, such as bowel involvement—as well as the surgical experience.

Although the tumours in the RA‐RTR cohort appeared significantly larger on preoperative imaging compared to those in the RA‐RPLND group, no significant difference was observed in the final specimen dimensions. This can be explained by the fact that RPLND is performed using a template‐based dissection in which the surgical specimen encompasses a wider anatomical area than the suspicious lymph node alone, resulting in larger resected specimens despite smaller preoperative tumour sizes.

Our RA‐RPLND cohort demonstrated perioperative outcomes comparable to the contemporary robotic series. Estimated blood loss was low (median 40 mL) and the median length of stay short (1 day), favourable relative to recently published multicentre cohorts (EBL 75–150 mL; LOS 2–3 days). No conversions to open surgery occurred in our series versus 3%–9% reported, and the overall complication rate (with no major events) was in line with prior data (6%–17%). The median operative time was 143 min, compared with 134–550 min in contemporary series.^8^, ^9^

Current guidelines recommend an open approach as the standard for the surgical management of retroperitoneal sarcomas.^10^ However, an increasing number of recent series have reported the feasibility and safety of RA procedures in this context.^3^, ^4^, ^5^ A broad range of histopathological subtypes was represented in the RA‐RTR cohort, including sarcomas, vascular, neurogenic, lymphatic, fibroblastic and sex cord–stromal tumours. Compared with other robotic series, our RA‐RTR cohort demonstrated low estimated blood loss (median 20 mL) and a short hospital stay (median 2 days), comparing favourably with published reports (EBL 30–192 mL; LOS 3.4–7 days) despite similar lesion sizes (median 56 mm vs. 46–75 mm). Conversion to open surgery was 0% in our series compared with up to 17% in other cohorts. The median operative time was 90 min, notably shorter than the 135–200 min described in contemporary series. The overall complication rate (5.6%) was within the range reported in the literature (approximately 1%–15%), further supporting the safety of this approach.^3^, ^4^, ^5^

This study is limited by its retrospective design and small cohort size, which reduce statistical power and limit generalisability. These constraints reflect the rarity and complexity of these tumours. The outcomes reported in this study may not be readily replicable outside highly specialised centres. The majority of procedures were performed by surgeons with extensive experience in complex open and robotic retroperitoneal surgery, within a high‐volume institution supported by dedicated multidisciplinary expertise. As such, these results likely reflect a combination of individual surgeon proficiency, institutional pathways and careful patient selection, rather than outcomes that can be assumed to translate to lower‐volume or less experienced settings.

Guidelines for both retroperitoneal tumours and testicular cancer recommend treatment in high‐volume multidisciplinary centres, reflecting the surgical expertise required for these complex procedures.^1^, ^10^ More than the choice between open or robotic approaches, it is surgical proficiency that determines outcomes. Naturally, RA procedures represent an evolution of the classical open technique and should likewise be performed in specialised reference centres.

Comparable perioperative outcomes between RA‐RTR and RA‐RPLND in our series suggest that, within specialised centres, urologists with established expertise in complex retroperitoneal surgery can extend robotic retroperitoneal techniques beyond conventional urological indications.

## AUTHOR CONTRIBUTIONS

All authors contributed to the study conception and design, material preparation, data collection analysis and writing of the manuscript. All authors read and approved the final manuscript.

## CONFLICT OF INTEREST STATEMENT

No disclosures.
